# An approach to the effects of longevity, sexual maturity, and reproduction on telomere length and oxidative stress in different Psittacidae species

**DOI:** 10.3389/fgene.2023.1156730

**Published:** 2023-03-20

**Authors:** Angélica Domínguez-de-Barros, Inés Sifaoui, Zuzanna Borecka, Roberto Dorta-Guerra, Jacob Lorenzo-Morales, Rafael Castro-Fuentes, Elizabeth Córdoba-Lanús

**Affiliations:** ^1^ Instituto Universitario de Enfermedades Tropicales y Salud Pública de Canarias (IUETSPC), Universidad de La Laguna, La Laguna, Tenerife, Spain; ^2^ Faculty of Biology and Animal Science, Wroclaw University of Environmental and Life Sciences, Wrocław, Poland; ^3^ Departamento de Matemáticas, Estadística e Investigación Operativa, Facultad de Ciencias Sección de Matemáticas, Universidad de La Laguna, La Laguna, Tenerife, Spain; ^4^ Departamento de Obstetricia y Ginecología, Pediatría, Medicina Preventiva y Salud Pública, Toxicología, Medicina Legal y Forense y Parasitología, Facultad de Ciencias de la Salud, Sección Medicina, Universidad de La Laguna, La Laguna, Tenerife, Spain; ^5^ Centro de Investigación Biomédica en Red de Enfermedades Infecciosas (CIBERINFEC), Instituto de Salud Carlos III, Madrid, Spain; ^6^ Departamento de Ciencias Médicas Básicas, Facultad de Ciencias de la Salud-Sección Medicina, Universidad de La Laguna, La Laguna, Tenerife, Spain

**Keywords:** aging, longevity, birds, telomeres, oxidative stress, breeding

## Abstract

**Introduction:** Aging is a multifactorial process that includes molecular changes such as telomere shortening. Telomeres shorten progressively with age in vertebrates, and their shortening rate has a significant role in determining the lifespan of a species. However, DNA loss can be enhanced by oxidative stress. The need for novel animal models has recently emerged as a tool to gather more information about the human aging process. Birds live longer than other mammals of the same size, and Psittacidae species are the most persevering of them, due to special key traits.

**Methods:** We aimed to determine telomere length by qPCR, and oxidative stress status using colorimetric and fluorescence methods in different species of the order Psittaciformes with different lifespans.

**Results:** We found that telomeres shorten with age for both long- and short-lived birds (*p* < 0.001 and *p* = 0.004, respectively), with long-lived birds presenting longer telomeres than short-lived ones (*p* = 0.001). In addition, short-lived birds accumulated more oxidative stress products than long-lived birds (*p* = 0.013), who showed a better antioxidant capacity (*p* < 0.001). Breeding was found related to telomere shortening in all species (*p* < 0.001 and *p* = 0.003 for long- and short-lived birds). Short-lived birds, especially breeding females, increased their oxidative stress products when breeding (*p* = 0.021), whereas long-lived birds showed greater resistance and even increased their antioxidant capacity (*p* = 0.002).

**Conclusion:** In conclusion, the relationship between age and telomere length in Psittacidae was verified. The influence of breeding increased cumulative oxidative damage in short-lived species, while long-lived species may counteract this damage.

## Introduction

The main diseases and causes of death in our actual society, such as cancer, neurodegenerative and cardiovascular diseases, are chronic and age-related conditions that still do not count with effective treatments (World Health Organization, 2019). A proposed approach to prevent or treat these conditions is to elucidate the mechanisms that lead to their outcome: aging.

Different theories exist to explain the process of aging and can be grouped into “evolutionary, molecular and cellular theories”, which are not mutually exclusive and can occur simultaneously (Weinert and Timiras, 2003). Telomere attrition is one of the “hallmarks of aging” proposed by López-Otín et al. (2013; 2023), which may explain the process of aging.

Telomeres are chromatin structures at the ends of linear chromosomes that consist of short DNA sequence repetitions (TTAGGG) with a t-loop structure. Telomeres protect chromosome ends from the fusion of two different double-stranded chromosome breaks (Nussey et al., 2014). These repeats are generated by a reverse transcriptase known as telomerase or “Tert”. Telomerase is more highly expressed in stem cells than in the rest of the somatic cells of multicellular organisms. Therefore, telomere sequence shortening occurs after each cell division due to the inability of DNA polymerase to completely replicate the terminal end of a linear strand (Levy et al., 1992; Blasco, 2007). In the absence of repair mechanisms, telomere length can be shortened to a critical length, triggering cellular senescence or apoptosis.

DNA loss can be enhanced by other factors, particularly oxidative stress. Excessive generation and accumulation of reactive oxygen species (ROS) and free radicals (FR) can damage major biomolecules, such as DNA, lipids, and proteins. (Mayne, 2003; García, 2005; Nussey et al., 2014).

The vital role of telomere length has been pointed out in aging processes and human diseases, such as cancer (Blackburn, 2005; Shen et al., 2007; McNally et al., 2019), respiratory illness (Jang et al., 2008; Celli, 2012; Córdoba-Lanús et al., 2018), cardiovascular diseases (Starr et al., 2007; Huzen et al., 2010; Terai et al., 2013), diabetes (Cheng et al., 2021), etc. Telomere length decreases progressively with age in many vertebrates, and its shortening rate could determine a species’ lifespan, which also depends on the different species (Vera et al., 2012; Whittemore et al., 2019). Moreover, telomere measurement provides important information by measuring chronic oxidative stress (Finkel and Holbrook, 2000). In humans, both environmentally induced and perceived stress shorten telomeres, consequently impacting longevity, health, and disease states (Epel et al., 2004). Animal modeling is used in science to understand this human process. In this matter, studying birds as aging animal models has gained ground recently (Holmes and Ottinger, 2003; Munshi-South and Wilkinson, 2009; Montgomery, 2011; Hickey et al., 2012; Holtze et al., 2021), and telomere shortening has been used as a tool to measure health, fitness, stress impact, reproductive success, and longevity of avian species (Swanberg, 2018). Birds tend to live longer and age slower than other mammals of the same size showing a Hamiltonian aging profile (Cohen, 2018). The avian class shows special physiological characteristics: a) Higher metabolic rate, b) Corporal temperature of 42°C, c) Higher levels of blood sugar and d) Highest pulmonary partial pressure of oxygen in all vertebrate groups (Holmes and Austad, 1995; Travin and Feniouk, 2016). Despite being predisposed to an increased generation of ROS, their long lifespan suggests that birds might be resistant to aging processes. Psittaciformes, including macaws, parrots, and related forms (parakeets) are found to be the most persevering birds, probably as a product of key traits such as delayed reproduction, heavy investment in reduced offspring, large brains, vocal communication, and social information transfer, which reduce extrinsic mortality and thus increase longevity (Brauth et al., 1997; Møller, 2006; McDonald Kinkaid, 2015; Smeele et al., 2022). Therefore, there is reason to expect that aging is systematically different in long-lived and short-lived species, and long-lived birds might reveal a novel model for studying human aging.

Studies carried out up to date and that we are aware of have compared telomere length and oxidative stress between different animal classes (Ku and Sohal, 1993; Herrero and Barja, 1999; Pamplona et al., 2005; Montgomery, 2011; Whittemore et al., 2019) or families (Hall et al., 2004; Montgomery et al., 2012; Tricola et al., 2018). However, there is a lack of such studies in psittacine birds with different biological ages and longevity strategies.

This cross-sectional study aims, firstly, to determine the telomere length and oxidative stress status of different species of the order Psittaciformes with different lifespans. Secondly, to establish the effect of certain factors, such as breeding, that may have on these biological markers in the life of birds. The animals in this study were captive birds belonging to the Breeding Centre of the Loro Parque Fundación Facilities, Loro Parque, Santa Cruz de Tenerife, Spain, which holds the largest genetic reserve of parrots in the world, where individuals are kept in optimal health conditions and there are no biases of external factors on life expectancy, such as predation, foraging, or extreme environmental factors.

## Materials and methods

### Individuals of study

A total of 81 psittacine birds of different species and longevities were analyzed: Forty-seven belonged to the classified long-lived group (3–36 years old) and the remaining 34 were short-lived birds (1–9 years old) (Table 1). As species selection criteria for the study, the register of the last 50 years of Loro Parque Fundación was considered, and a database/literature search was carried out on the maximum expectation of life of Psittacidae species. In addition, the similarity in body size, diet administered, and sample range available at the facilities were considered. A further subdivision of the individuals according to their similar age at sexual maturation was made, as it marks an important milestone in their life history that may have consequences on the parameters of the study (Table 2).

**TABLE 1:** Characteristics of long-lived and short-lived species of Psittacidae included in this study.

Group	Species	N	Lifespan (years)^a^	Age (years)^b^	Body mass (g)^c^
**Long-lived species**	*Amazona barbadensis*	22	35	13 (3–29)	309.31
	*Anodorhynchus hyacinthinus*	8	38	29 (23–33)	1552.33
	*Cacatua moluccensis*	9	65	25 (9–35)	880.89
	*Ara macao*	8	33	20 (6–36)	1027.71
**Short- lived species**	*Agapornis taranta*	8	15	7 (1–7)	47.33
	*Psitteuteles goldiei*	19	10	1 (1–7)	52.07
	*Trichoglossus johnstoniae*	7	17	7 (2–9)	58.16

^a^ Life expectancy data and breed information from AnAge database (https://genomics.senescence.info/species/index.html) and ZIMS Species 360 Global Information Serving Conservation database (https://zims.species360.org/Login.aspx?ReturnUrl=%2f).

^b^ Age: median (min-max).

^c^ Average of body mass (g).

**TABLE 2:** Distribution of individuals in breeding groups according to the age at which they reach sexual maturity.

**Group**	**Species**	**Breeding age (yrs)** ^a^		**Breeding group**				
				**Immature** (<10 years) **N**		**Young** (10–20 years) **N**		**Adult** (>20 years) **N**
**Long-lived species**	*Amazona barbadensis*	5		9^b^		9		4
	*Anodorhynchus hyacinthinus*	10		—		—		8
	*Cacatua moluccensis*	10		1		2		6
	*Ara macao*	10		3		1		4

				**Immature** (<3 years) **N**		**Young** (3–5 years) **N**		**Adult** (>5 years) **N**
**Short-lived species**	*Agapornis taranta*	2		2		—		6
	*Psitteuteles goldiei*	2		16		—		3
	*Trichoglossus johnstoniae*	3		1		—		6

^a^ Age at which the individuals reach sexual maturity according to AnAge database (https://genomics.senescence.info/species/index.html).

^b^ For *Amazona barbadensis* immature individuals were all aged <5 years.

### Sample collection

Birds are kept in cages differentiated by species. The juvenile individuals are placed in community aviaries, being able to coexist with other species when required, and adult individuals are separated into breeding couples. Sampling was done once the breeding season was over, so as not to increase stress situations, taking place in September for short-lived birds, and November for long-lived ones.

Each individual underwent blood extraction by venipuncture from the right jugular vein to perform the pertinent analysis detailed below. Blood samples were stored at <5°C for up to 2 h upon centrifugation at 3,000 rpm for 10 min to separate the plasma from red blood cells. Biological samples have been taken as part of the routine annual veterinary control without any harm or risk to the life of the animals under study. Animals were treated appropriately according to the Spanish legislation and the EU Directive (2010/63/UE) on “Protection of Animals Used for Experimental and Other Scientific Purposes”. The study complies with the ARRIVE guidelines developed by the NC3Rs, and all efforts were made to minimize the number of animals used to produce reliable scientific data, as well as animal suffering.

### Telomere length measurement

DNA was extracted from 5 µL of whole blood using the Dneasy Blood & Tissue Handbook kit (Qiagen) following the instructions of the manufacturer. The DNA concentration and purity were quantified using a NanoDrop Lite Spectrophotometer (Thermo Scientific). Optimal purification criteria were set up on 1.80 at 260/280 nm.

Telomere length was assessed using the real-time PCR (qPCR) procedure first described for humans by Cawthon (2002) and later adapted to birds by Criscuolo et al. (2009). Briefly, this method is based on the determination of amplification cycles necessary to detect a lower threshold of fluorescent signal, with the cycle number (CT) being proportional to the telomere length (T), or to the number of copies of a control gene (S). A ratio (T⁄S) is then calculated for each sample that will reflect relative inter and intra-individual differences in telomere length. To obtain a relative T/S ratio, the formula $\frac{T}{S}=2^{\left(-∆CT\right)}$ was used and corrected by the method proposed by Pfaffl (2001) to normalize the variation in amplification efficiencies.

The control gene used in the reaction was glyceraldehyde-3-phosphate dehydrogenase (***GAPDH***), (Fw-5′ GTGGTGCTAAGCGTGTTATCATC-3`//RV-5′ GGC​AGC​ACC​TCT​GCC​ATC3′) and the specific primers for telomere length amplification (***TEL*)** were as follows: (Fw-5′ CGGTTTGTTTGGGTTTGGGTTTGGGTTTGGGGTTTGGGTT-3’//RV-5′ GGC​TTG​CCT​TAC​CCT​TAC​CCT​TAC​CCT​TAC​CCT​TAC​CCT-3’). qPCR for both, *GAPDH* and *TEL*, was performed with 200 nM of primers and 1.25 ng of DNA per reaction in a final volume of 10 µL containing 5 µL of Power SYBR^®^ Green PCR Master Mix (Applied Biosystems). A reference sample belonging to the species *Anodorhynchus hyacinthinus* was used, from which a standard curve was created using serial dilutions at the following concentrations: 40 ng/μL, 10 ng/μL, 2.5 ng/μL, and 0.6 ng/μL. The reference sample was used as an internal control in each qPCR plate (telomeres and control gene independently) to control for the amplification efficiency of the qPCR and to set the threshold Ct value for both long- and short-longevity birds. Differences between the original protocol and the used one were established using the chosen individual as a reference sample for all species included in the study.

*GAPDH* and *TEL* amplifications were performed independently in a StepOnePlus thermocycler (Applied Biosystems, ThermoFisher Scientific, United States). *GAPDH* PCR conditions were as follows: 10 min at 95°C, followed by 40 cycles of 15 s at 95°C, 30 s at 56°C, and 30 s at 72°C. The *TEL* PCR conditions were 10 min at 95°C followed by 27 cycles of 15 s at 95°C, 30 s at 56°C and 30 s at 72°C. All samples were run in duplicate. Intra-plate coefficients of variance (CV) were calculated between the replicates and samples with CV > 5% were excluded from further analysis. Inter-plate CV for the calibrator sample was calculated to be <7%.

### Quantification of the total antioxidant capacity (TAC)

The measurement of antioxidant capacity was based on the reduction of ferric ion (Fe^3+^) when combined with a radical such as TPTZ (tripyridyl-s-triazine), turning into a ferrous ion (Fe^2+^). The Fe^2+^ concentration in serum samples was determined by preparing a working solution containing Acetate Buffer (30 mM), Iron Trichloride (20 mM) and 2, 3, 5-Triphenyltetrazolium chloride (TPTZ) (10 mM) in a 10:1:1 ratio. 10 μL of serum samples were used per reaction. The reaction was measured by spectrophotometry and absorbance was quantified at 593 nm using an EnSpire Multimode Plate Reader (Perkin Elmer, Madrid, Spain). Equivalent units of iron (µM Trolox) were quantified using the standard curve of Trolox (Total Antioxidant Capacity Assay Kit, Abnova ™). Each sample was evaluated in duplicate.

### Quantification of lipid peroxidation products by the TBARS assay

The thiobarbituric acid reactive substance assay (TBARS) was used to determine the level of malondialdehyde (MDA), the major lipid oxidation product, as well as some minor related compounds of lipid peroxidation in serum samples. The assay was performed using the OxiSelect TBARS Assay Kit (Cell Biolabs, Inc. United States) following the instructions of the manufacturer. The MDA-TBA adduct formed was measured fluorometrically at 540 nm excitation and 592 nm emission wavelength on an EnSpire Multimode Plate Reader (Perkin Elmer, Madrid, Spain). Quantification of MDA (µM) was quantified using a Standard Curve. Each sample was evaluated in duplicate.

### Statistical analysis

Results are presented as means ± standard deviation (SD) for continuous data and proportions for categorical data. The data obtained were tested in their distribution for normality by Shapiro-Wilk’s test or Kolmogorov-Smirnov goodness-of-fit test, as appropriate. Homogeneity of variances was assessed by Levene’s test for equality of variances. The correlation between variables was assessed using Pearson´s product-moment coefficient when variables were bivariate normally distributed and Spearman’s rank-order correlation when normality was not achieved. The variable “age” was standardized for further analysis since the passing of 1 year of age is not the same for the different species within each longevity strategy.

ANOVA and ANCOVA tests were used to compare the study factors on the variables of interest. When an ANCOVA was applied, the linear relationship between standardized age and response variable for each group combination was assessed by visual inspection of the response variable vs. covariate scatterplot, and homogeneity of regression slopes was assessed by the no significance of interaction term. When a statistically significant interaction was detected, the two or three factors, as appropriate, were evaluated simultaneously by the estimated marginal means plots for all the levels of each single factor and the simple pairwise comparisons were run with a Bonferroni adjustment. Alternatively, if non-statistically significant interaction was verified, the means were compared using Tukey’s honest significant difference multiple comparison test to evaluate the effects of factors. Statistical analysis was performed using SPSS v.25.0 (IBM Statistics) and the accepted level of significance was *p* < 0.05. Graphs were designed with GraphPad Prism v9.0 (Dotmatics, GraphPad Software, San Diego, California United States).

## Results

### Telomere length

**FIGURE 1:**
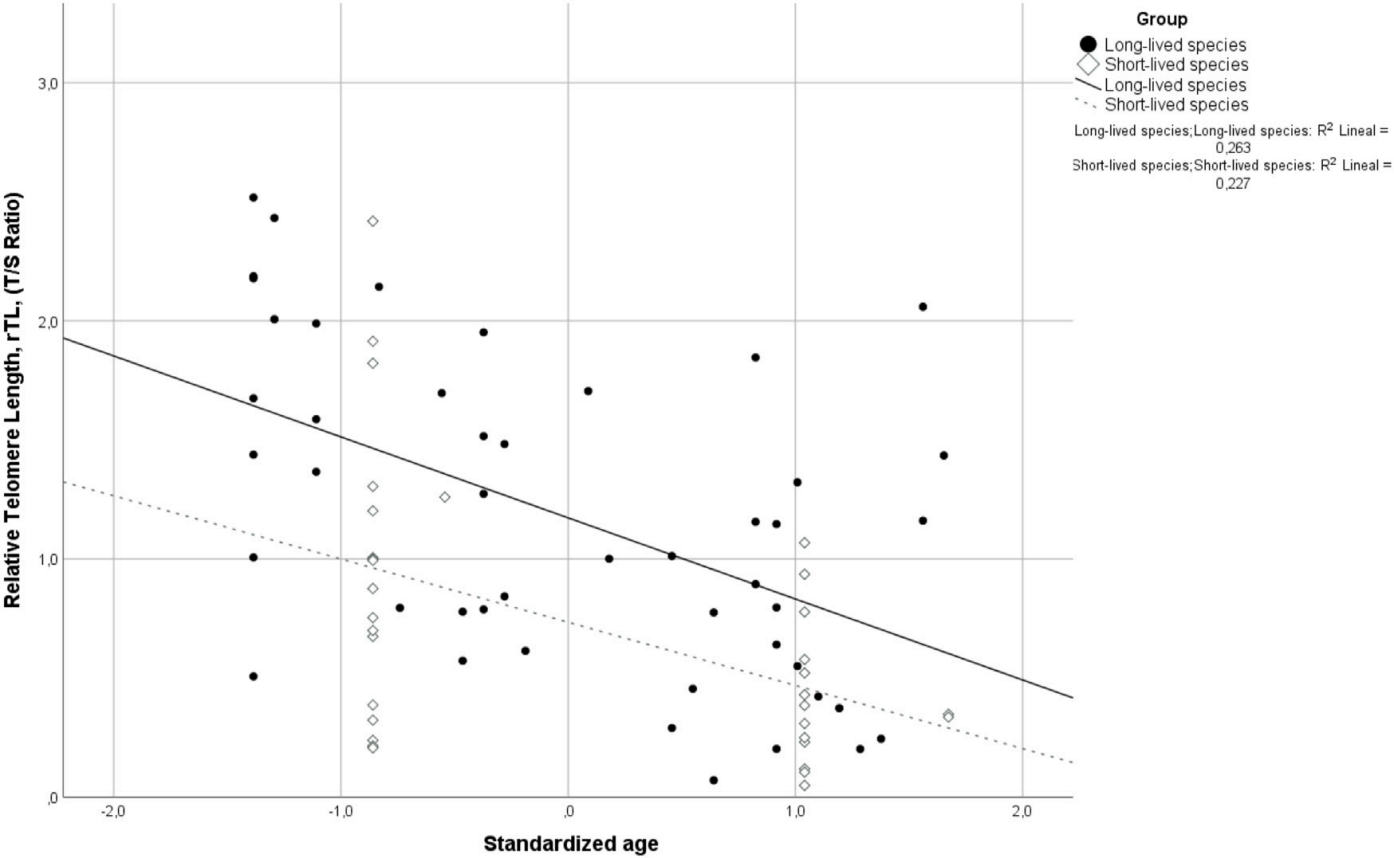
Correlation between relative telomere length (rTL) and standardized age for long- and short-lived birds. Spearman’s coefficient correlation (R = −0.472, *p* < 0.001) includes both long- and short-lived birds.

Relative telomere length (rTL) was inversely correlated with age in both long-lived (R = −0.491; *p* < 0.001) and short-lived birds (R = −0.428; *p* = 0.012) (Figure 1). Since then, subsequent analyses were performed by adjusting for the effect of age.

Long-lived birds presented longer rTL than short-lived ones (*p* < 0.001), with the rTL of long-lived birds being 0.629 units longer than that of short-lived ones. The relative telomere length was found to be independent of the sex (*p* = 0.825).

**FIGURE 2:**
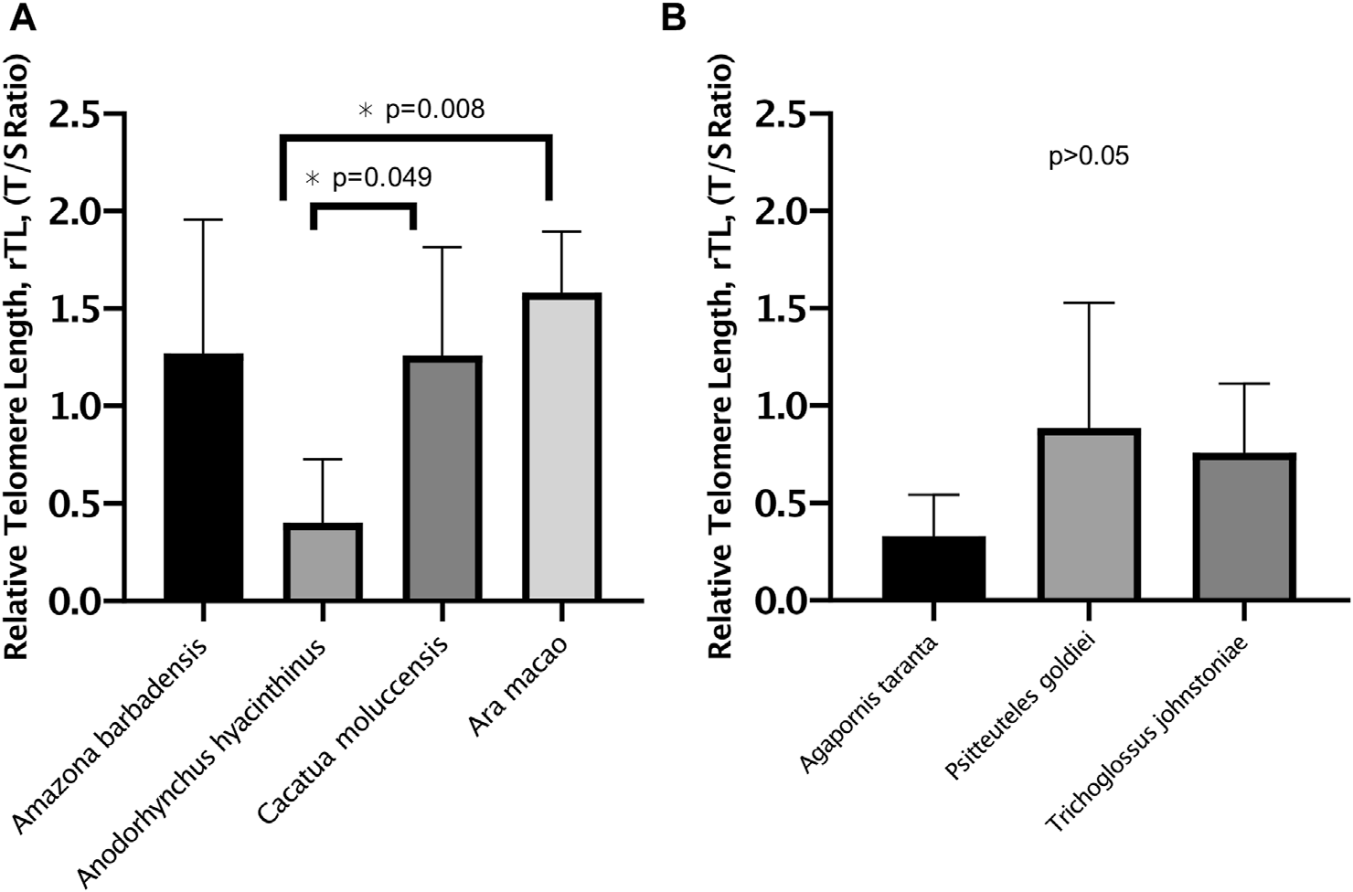
Relative telomere length (rTL) of Psittacidae species. **(A)** rTL (mean ± SD) of long-lived species. **(B)** rTL (mean ± SD) of short-lived species. Assessed by one-way ANCOVA controlling for standardized age.

When analyzing rTL between species within the different longevity groups, telomere length differed between the long-lived species (*p* = 0.005) (Figure 2A). *Anodorhynchus hyacinthinus* had the shortest rTL, with 0.704 telomere units shorter than *Cacatua moluccensis* (*p* = 0.049) and 0.921 telomere units shorter than *Ara macao* (*p* = 0.008). However, we found a similar rTL distribution between the species of short-lived birds (*p* > 0.05) (Figure 2B).

Reaching sexual maturity was found to be an influential factor for telomere length in long-lived birds (*p* = 0.015). Immature individuals had longer rTL than both young mature (*p* = 0.005) and mature adults (*p* = 0.006). We found no influence of maturity in rTL on short-lived birds (*p* = 0.666) (Table 3).

**TABLE 3:** Relative telomere length at different maturity status of the different longevity ^**‡**^Groups at standardized age.

Group^‡^	Maturity status	N	Telomere length^a^	*p*-value^b^
**Long-lived species**	Immature	13	2.258 ± 0.372	
	Young	12	1.308 ± 0.183	**0.005**
	Mature	22	0.457 ± 0.280	**0.006**
**Short-lived species**	Immature	19	0.945 ± 0.506	
	Mature	15	0.453 ± 0.638	0.666

^a^Mean rTL ± S.E.

^b^p-values < 0.05 are represented in bold.

**FIGURE 3:**
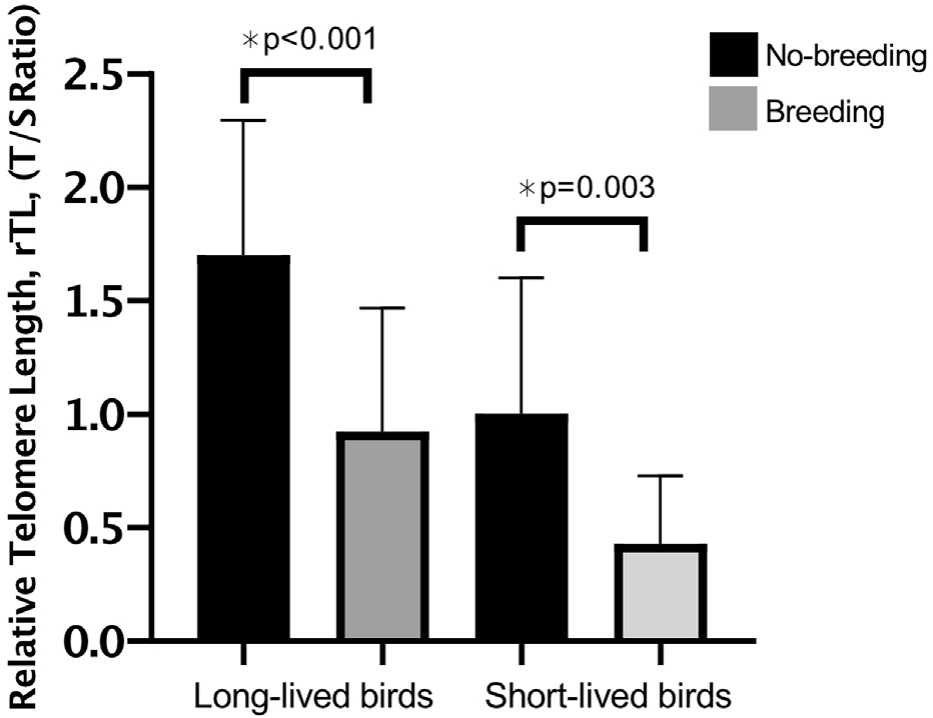
Relative telomere length (rTL) in long- and short-lived birds with different breeding statuses. Mean rtL ±SD of breeding vs. non-breeding for long- and short-lived birds. A three-way ANOVA was conducted to determine the effects of sex, longevity and reproduction. No interactions were statistically significant. There was an effect of longevity and reproduction on rTL, however, the differences in rTL for male and female birds were not statistically significant.

Our results suggest that breeding negatively influences telomere length (1.332 ± 0.11 in breeders vs. 0.882 ± 0.09 in non-reproductive birds; *p* < 0.001). Both, long- and short-longevity birds that had bred presented shorter telomeres than non-reproductive birds (*p* < 0.001 and *p* = 0.003, respectively) (Figure 3).

### Oxidative stress

**FIGURE 4:**
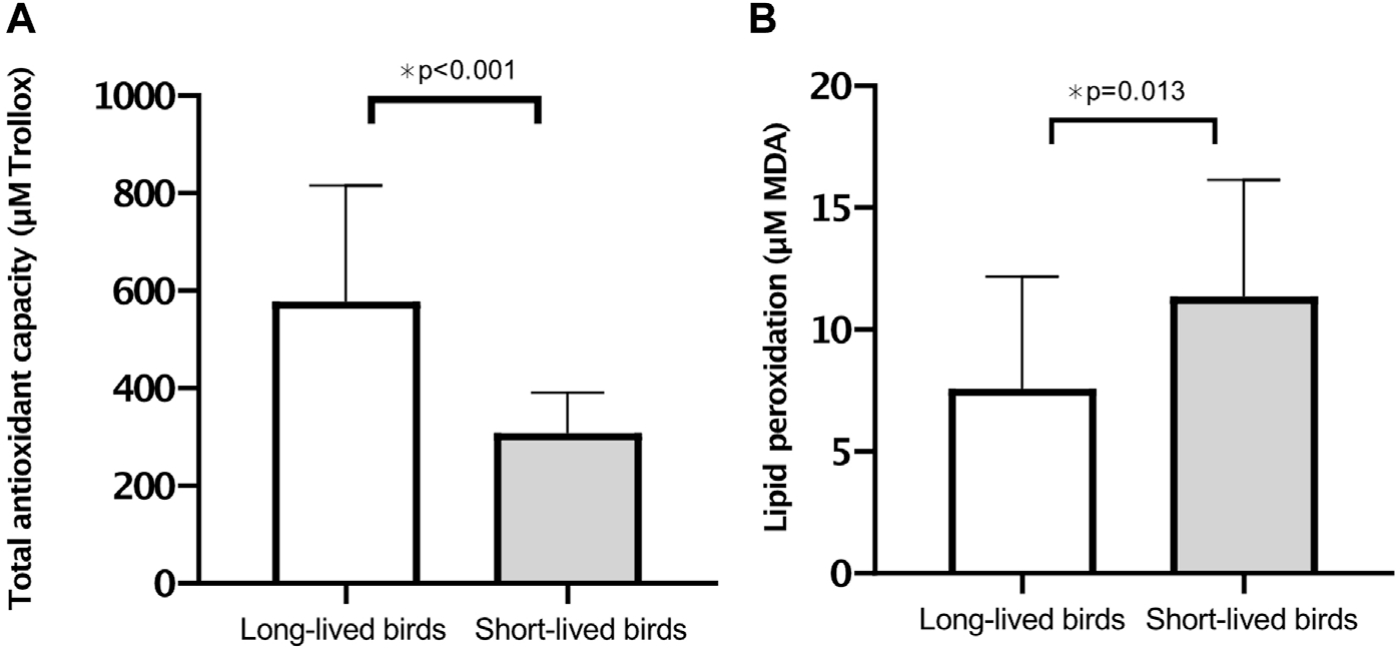
Markers of oxidative stress. **(A)** Total antioxidant capacity (mean of µM Trolox equivalents ±SD) in long-vs. short-lived psittacine. **(B)** Lipid peroxidation products (mean of µM MDA ±SD) in long-vs. short-lived psittacine. A two-way ANOVA was conducted to determine the effects of longevity and sex.

The oxidative stress analysis revealed that long-lived birds had greater antioxidant capacity than short-lived birds (*p* < 0.001) (Figure 4A), with no sex distinction (*p* = 0.665). In contrast, short-lived birds had increased levels of lipid peroxidation products than long-lived birds (*p* = 0.013) (Figure 4B). Furthermore, both long- and short-lived females had higher levels of TBARS than males (13.77 ± 1.18 vs. 7.06 ± 1.21, respectively; *p* = 0.012).

**FIGURE 5:**
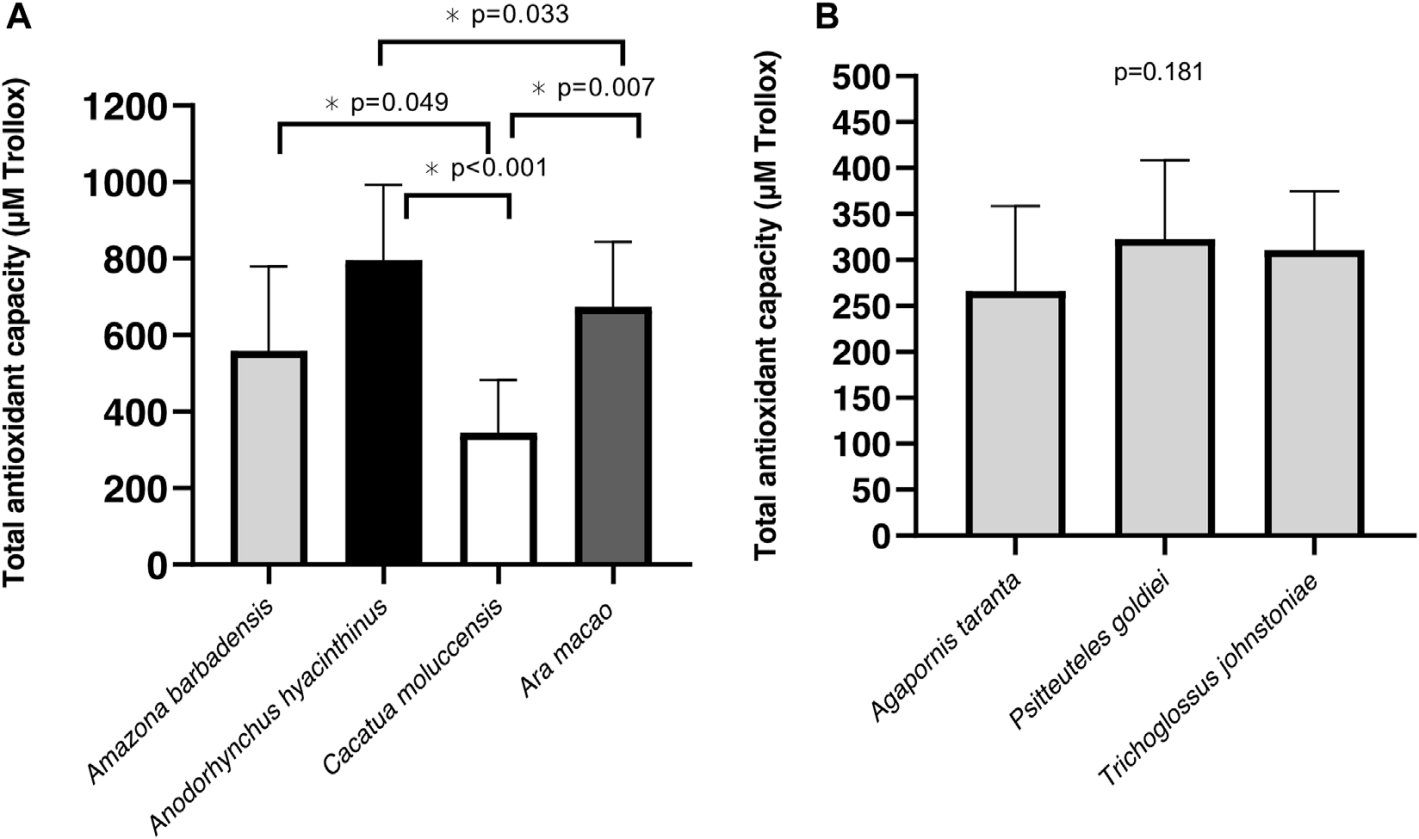
Total antioxidant capacity (TAC) of Psittacidae species. **(A)** TAC levels in long-lived species **(B)** TAC levels in short-lived species. Mean of µM Trolox equivalents ±SD, assessed by one-way ANOVA to determine the effects of species for long- and short-lived Psittacidae.

Antioxidant capacity was significantly different within species of the long-longevity group (*p* < 0.001). *Cacatua moluccensis* had the lowest antioxidant capacity of the species. On the other hand, the two species with the highest TAC were *Anodorhynchus hyacinthinus* and *Ara macao*, respectively **(** Figure 5A **)**. We did not find any differences in TAC between the species of short-lived birds (*p* = 0.181) (Figure 5B). When measuring TBARS within the different species of each group, the long-lived species showed similar levels between them (*p* = 0.130).

The state of sexual maturity did not involve significant changes in the levels of TAC compared to immature individuals, neither for long- (629.41 ± 49.59; 572.50 ± 76.20 and 494.53 ± 59.08 for mature, young mature, and immature, respectively; *p* = 0.272), nor for short-lived birds (282.73 ± 16.80 for mature vs. 325.95 ± 21.64 for immature; *p* = 0.141). Conversely, birds underwent changes in their lipid oxidative damage as they entered a mature state. In the long-lived group, young mature birds had higher TBARS levels than mature adult birds (10.39 ± 0.77 vs. 5.31 ± 0.63, respectively; *p* = 0.012). Between short-lived individuals, mature birds had higher levels of TBARS than immature ones [34.93 (12.73–107.92) vs. 9.65 (5.84–11.93), respectively; *p* = 0.038].

**FIGURE 6:**
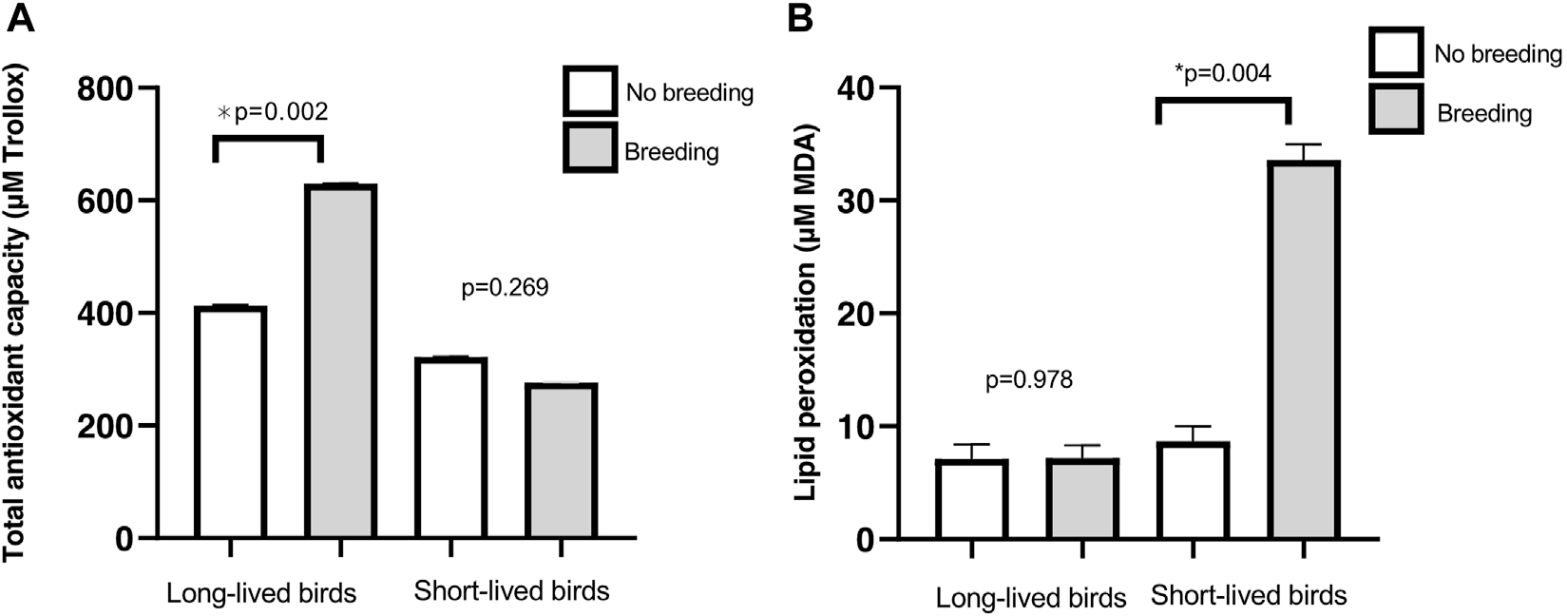
Markers of oxidative stress in relation to reproduction. **(A)** Total antioxidant capacity (mean of µM Trolox equivalents ±SD) in long- and short-lived birds with different breeding status. A three-way ANOVA was conducted to determine the effects of sex, longevity and reproduction. **(B)** Lipid peroxidation products (mean of µM MDA ± SD) in long- and short-lived birds with different breeding status, conducted by a two-way ANOVA.

When we studied the effects of reproduction, long-lived birds had increased antioxidant capacity after breeding than non-reproductive birds (*p* = 0.002). Moreover, there was no breeding effect on TAC in short-lived birds (*p* = 0.269) (Figure 6A). TBARS levels in reproductive-long-lived birds did not vary significantly from non-reproductive ones (*p* = 0.978), while in short-lived birds, TBARS levels were increased in individuals that had bred (*p* = 0.004) (Figure 6B **)**. If we focus only on females, those that had gone through the reproduction process had higher levels of TBARS (19.14 ± 1.25 vs. 7.50 ± 1.36, reproductive vs non-reproductive females respectively; *p* = 0.021), independent of their longevity (*p* = 0.088).

## Discussion

This study was performed to analyze the use of a set of biomarkers capable of evaluating in the most possible complete way the biological state, fitness, and strategies in Psittacidae in relation to aging.

### Telomere length

Our study is the first to study various species of well-defined groups of birds with different longevity (short- and long-lived birds) within Psittacidae in relation to telomere measurement and oxidate stress status. As expected, longer telomeres were found in long-lived birds when compared to short-lived ones. We also confirmed that both groups lost telomere repetitions with increasing age, in agreement with other studies reporting telomere shortening in different birds and other animal species (Haussmann et al., 2003a; Vera et al., 2012; Sudyka et al., 2016). Our findings also agree with Remot et al. (2020), who reported that telomere length did not differ between the sexes of different species of birds. Although we cannot rule out that differences could exist between sexes when other factors come into play, such as reproductive pressure, that may affect males and females differently, for which a larger sample should be studied.

Moreover, different telomere length distributions were observed between species in long-lived species. *Ara macao* and *Cacatua moluccensis* had the longest telomere length (rTL). For these species records of longevity in captivity have been documented: nearly 65 years for *Corsica Macaw* (Ara) and 69 years for *King Tut* (Cacatua), respectively (Brouwer et al., 2000). It is suggested that these species could have some telomere conservation mechanism that short-lived species lack hence leading to a faster loss of telomeric repeats throughout their lives, affecting their longevity (Haussmann et al., 2003b).

Telomere length varied at different stages of maturity in long-lived birds, where immature individuals presented longer telomeres. We could observe that the age of sexual maturity in long-lived birds under study is around 10 years. Delaying sexual maturity may be an evolutionary trait that has been positively selected, and according to Skulachev et al. (2017), developmental delay leads to the retardation of aging. Shorter telomeres were found in birds after the breeding period which is supported by Beaulieu et al. (2011) study, who conclude that “organisms may be constrained to reallocate resources under specific circumstances to a given function at the expense of another one”. Both longevity groups ended up losing telomeres when breeding as this is a stressful and energy-demanding process as demonstrated by Bauch et al. (2013) and Reichert (2013). But the question would be whether birds with high longevity manage to lose fewer telomeric repeats than those with short longevity. The answer might be through some protective mechanism such as antioxidant capacity. This justifies that long-lived birds tend to self-maintain, in contrast to short-lived birds. However, unraveling this would require a longitudinal study. Studying telomere length variation as a biomarker of individual quality could represent a promising framework for exploring the cost of reproduction (Sudyka, 2019).

### Oxidative stress

Long-lived birds tend to have greater antioxidant capacity at an increased age, which may imply that they use this mechanism when faced to the loss of telomeric repeats. In the same way, lipid peroxidation products were increased in aged, short-lived birds. However, we cannot conclude that these relationships are causal, a longitudinal study with larger sample size is required. Literature data on age-related changes in oxidative lipid damage have been inconsistent, probably because most of the research carried out in this field surveyed only one species or compared diverse taxa (Bize et al, 2014; Matsuo et al., 1993).

Some comparative studies have reported an inverse relationship between oxidative damage and longevity; specifically, long-lived birds showed less oxidative damage than similar-sized but shorter-living mammals (Pamplona et al., 1996). We confirmed these results by finding more lipid peroxidation compounds in short-lived birds, a proposed biomarker of oxidative damage. However, there is a certain contradiction when comparing lipid peroxidation damage between species with different longevities. The study by Montgomery et al. (2012), found no differences in TBARS levels between short-lived quails and long-lived parrots. Although, this is the only research we know to compare lipid peroxidation damage between birds of different longevities, it did not study the cited marker within Psittaciformes. Differences in longevity may be explained by how these mechanisms evolve in each species. Natural selection promotes longevity in long-lived animals as they have a lower production of mitochondrial ROS, and their specific composition of tissue macromolecules (proteins, lipids, and nucleic acids) gives them an intrinsically high resistance to modification which likely contributes to their superior longevity (Pamplona and Barja, 2011). Our results that showed that short-lived parrots are more susceptible to damage, and therefore accumulate more oxidative products than long-lived birds due to their configuration of membrane lipids and proteins are in line with these conclusions and in agreement with Galván et al. (2015).

In addition, the differences in antioxidant capacity between long-lived species may indicate that specific mechanisms are selected to maintain longevity. *Anodorhynchus hyacinthinus*, followed by *Ara macao*, are the species with the highest TAC levels, being the first and third oldest birds in the long-lived group (mean age 29 and 20 years, respectively). However, these species differ in their telomeric length. While *Anodorhynchus hyacinthinus* had the shortest rTL of its group, *Ara macao* had the longest telomeres. This may indicate that the antioxidant capacity in *Anodorhynchus hyacinthinus* is a mechanism used either as protection or as a response to oxidative damage, but in *Ara macao,* it may imply that this mechanism plays an important role in protecting its telomeric sequence. As we previously mentioned, in agreement with Brouwer et al. (2000), cases of extreme longevity have been reported for *Ara macao*. To the best of our knowledge, characterization of the antioxidant capacity status and its possible relationship with longevity in these species without dietary supplementation experiments have not been performed.

We could not find conclusive results on changes in antioxidant capacity at the different stages of sexual maturity for any of the studied species. However, we did find that young mature individuals within long-lived birds had increased levels of TBARS products compared to older mature individuals. Similarly, in short-lived birds, mature individuals had a higher level of lipid peroxidation than immature individuals. Our results agree with those reported by Marasco et al. (2017), who found an increase in oxidative stress (measured by 8-OHdG and protein carbonyls) from early adulthood to mid-adult life in female zebra finches. In the same way, Alonso-Álvarez et al. (2010), reported that old partridges had higher levels of TBARS than middle-aged partridges (considering the partridge as a short-lived species with a life expectancy of 1–4 years).

Finally, our findings in relation to oxidative stress and reproduction suggest that this period may affect differently depending on the longevity strategy of these birds. While in short-lived birds there is an accumulation of lipid peroxidation, the opposite effect occurs in long-lived ones. Theoretically, reproduction is an expensive trait because it leads to an increased metabolic rate, which results in increased production of ROS and oxidative damage (Costantini, 2008; Monaghan et al., 2009). We found that short-lived species had higher levels of TBARS after breeding. The fact that levels of lipid peroxidation products remain stable in long-lived birds while TAC increased when breeding, may suggest that these birds had developed a balanced life strategy in favor of longevity that improves their capacity to carry out effective somatic maintenance in terms of oxidative homeostasis, in agreement with Vágási et al. (2019). This leads us to propose that a key to maintaining a high life expectancy in these birds lies in activating their defenses to counteract this possible damage, a capacity that does not exist or is not effectively functional in short-lived birds.

Although¸ there is still some controversy regarding the role of oxidative damage in reproduction. On one hand, Selman et al. (2012) found no association between reproductive effort and oxidative damage, however, Blount et al. (2016) found the opposite pattern than expected in a metanalysis, where a decrease in oxidative damage occurs in reproducers. In our study, after collecting data at the same time for all individuals and once the reproductive period had elapsed, we found that females that had bred, both short- and long-longevity ones, had higher levels of lipid peroxidation products than males and non-breeding females. Blount et al. (2016) suggested that reproduction could affect females more since oxidative stress during egg production has the potential to damage specific proteins. In addition, aldehydic lipid peroxidation products, such as malondialdehyde, are incorporated into the lipid matrix of the yolk, leading to oxidative degeneration of the yolk nutrients and lipid peroxidation *in vivo*.

Even so, we are aware that we observe an increase in oxidative stress that has only been studied in the lipid peroxidation pathway, and that a set of markers is necessary to comprehensively understand all the metabolic changes that occur in relation to reproduction and their affectation on the telomeric dynamics.

### Strengths of the study

Studying telomere dynamics and oxidative stress in birds may be advantageous for several reasons. Working with these types of animals, and especially due to the conservation work carried out by Loro Parque Fundación, we could obtain quantitative and qualitative data over time, making it possible to carry out future longitudinal studies that are important for the study of telomere dynamics. Vertebrate telomere length studies use blood as the work tissue, from which we can obtain the necessary amount of sample to carry out our experiments generally with little invasive effect. Moreover, birds have nucleated erythrocytes, allowing the measurement of telomere length in a tissue that is representative of the telomere lengths exhibited by other somatic tissues (Reichert, 2013).

Since there is controversy regarding how these types of experiments should be carried out to study either the effort made or the cost of reproduction on telomeric length or stress in birds, as reported by Metcalfe and Monaghan (2013), we want to point out that in this work, the reproduction period has not been manipulated, neither by making changes in the environment (temperature, abundance of food) nor in the pairs already formed; it has only been taken into account if the couple has succeeded in laying eggs.

### Limitations of the study

The possible problems derived from working with this type of animals are found in the availability of individuals at the time of blood extraction, because some are in the clinic facilities due to some disease or died due to their elevated age. In addition, since it is an observational study with a cross-sectional characterization of the state of these individuals, we may be missing the effects of the parameters. We are aware that when subdividing in species the number of individuals analyzed reduces, hence our results must be validated in a longitudinal study.

A limitation to point out is that in our analysis we did not control for phylogenetic relationships between species. Nevertheless, our interesting findings represent a starting point in studies on telomeres in psittacine species that must be validated in further prospective research with an adequate phylogenetic characterization on a wide sample of individuals.

In addition, we also systemically look at antioxidant systems, without knowing which pathways are activated (whether enzymatic or non-enzymatic), so we cannot establish causal relationships from trends in our results and longitudinal studies are necessary.

The use of animal models is essential to unravel the mechanisms of aging and facilitate the transfer of knowledge to the human aging process. The use of novel animal models provides us a different approach to the already well-known traditional mouse model. Although it is important to include non-traditional species in aging model organisms to integrate novel and relevant information, they must be used with caution, and their consequent results should be interpreted through a wide comparative framework.

In conclusion, in this study, we verified the inverse correlation between age and telomere length in Psittacidae and found that within different species, those with a long-lived strategy have a longer telomere length than those with short longevity. This strengthens the telomere length value as a possible biomarker of aging and its usefulness as a tool for measuring the individual state and life strategies of an organism. We have also described the influence of the reproductive process, the increase in oxidative stress levels, and its possible consequences on telomeric length loss and accumulated lipid damage, especially in species with low longevity. Finally, species with high longevity show greater resistance to oxidative damage during reproductive processes, even increasing their antioxidant capacity. Further studies of the longitudinal dynamics of these promising markers are warranted.

## Data Availability

The original contributions presented in the study are included in the article/supplementary material, further inquiries can be directed to the corresponding author.
